# A *PROMOTER::LUCIFERASE* reporter system reveals key elements of the circadian regulation of Crassulacean acid metabolism (CAM) in *Kalanchoë laxiflora* Baker

**DOI:** 10.1111/tpj.70937

**Published:** 2026-06-04

**Authors:** Jessica H. Pritchard, Jade L. Waller, Peter J. D. Gould, Nirja Kadu, Susanna F. Boxall, Louisa V. Dever, Jana Kneřová, Diarmuid O'Maoileidigh, James Hartwell

**Affiliations:** ^1^ Department of Biochemistry, Cell and Systems Biology Institute of Systems, Molecular and Integrative Biology, University of Liverpool Crown Street Liverpool L69 7ZB UK; ^2^ Present address: School of Biological Sciences Institute of Molecular Plant Sciences, University of Edinburgh Edinburgh EH9 3BF UK; ^3^ Present address: Farm Urban 401 Tower Street, Brunswick Business Centre Liverpool L3 4BJ UK; ^4^ Present address: Ceratium BV The Haven, West Kirby Wirral CH48 8AP UK; ^5^ Present address: Centre of Plant Structural and Functional Genomics Institute of Experimental Botany of the Czech Academy of Sciences 77900 Olomouc Czech Republic; ^6^ Present address: Department of Biology Maynooth University Maynooth County Kildare Ireland

**Keywords:** *Kalanchoë laxiflora* Baker, Crassulacean acid metabolism, plant circadian clock, firefly luciferase reporter gene, imaging analysis of luciferase circadian rhythms, gene promoter characterisation, leaf developmental regulation

## Abstract

Crassulacean acid metabolism (CAM) plants perform primary atmospheric CO_2_ fixation at night, with timekeeping by the endogenous circadian clock. Understanding of circadian coordination of CAM remains limited to rhythmic post‐translational regulation of phosphoenolpyruvate carboxylase (PPC) by a specific clock‐controlled protein kinase, PPCK. Here, candidate promoter regions (~3000 bp) of CAM‐associated genes from *Kalanchoë laxiflora* Baker were coupled to a firefly luciferase reporter and stable transgenic lines of both *K. laxiflora* and C_3_ *Arabidopsis thaliana* were generated. In *K. laxiflora,* the CAM‐associated *GLUCOSE 6‐PHOSPHATE/PHOSPHATE TRANSLOCATOR2* promoter (*KlGPT2p*) generated robust circadian rhythms of luciferase luminescence in constant conditions, with peak activity in leaf pair 3, where CAM‐associated nocturnal CO_2_ fixation initiated during leaf development. *KlGPT2p::LUC+* did not drive rhythms of luminescence in Arabidopsis and the *KlPPCK1* promoter produced no LUC+ signal in either species. Furthermore, the *CHLOROPHYLL A/B BINDING PROTEIN2* promoter (*KlCAB2p*), a clock‐controlled promoter that drives a gene involved in light‐reactions of photosynthesis, drove robust rhythms in both *K. laxiflora* and Arabidopsis. *KlCAB2p* circadian period changed during leaf development in *K. laxiflora,* and peak activity shifted from dusk in CAM leaves of *K. laxiflora* to dawn in Arabidopsis, highlighting differences in the timing of outputs from the core clock between species. These findings establish a robust *PROMOTER::LUC+* reporter system in a CAM plant, highlight divergent timing driving clock‐controlled promoters between species, and period lengthening with leaf age in *Kalanchoë*.

## Significance Statement

Circadian rhythms of firefly luciferase in the Crassulacean acid metabolism (CAM) species *Kalanchoë laxiflora* were driven by both CAM and non‐CAM gene promoters, revealing valuable new insights into both the leaf developmental and circadian clock control associated with CAM. The CAM *KlGPT2* promoter drove rhythms in *K. laxiflora*, but not *Arabidopsis thaliana,* whereas the non‐CAM *KlCAB2* promoter drove robust rhythms in both species with a peak phased to dawn in C_3_ Arabidopsis but dusk in *K. laxiflora*.

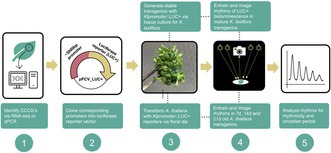

## INTRODUCTION

Crassulacean acid metabolism (CAM) requires fine‐tuned temporal control of metabolism to avoid futile cycling during CO_2_ assimilation (Boxall et al., 2017; Hartwell, 2006). While plants using C_3_ and C_4_ photosynthesis open their stomata to assimilate CO_2_ in the light period, CAM plants invert stomatal opening and shift primary atmospheric CO_2_ assimilation to the dark period when CO_2_ is fixed by phosphoenolpyruvate carboxylase (PEPC or, hereafter, PPC). This shift to opening stomata in the dark and closing them in the light leads to dramatic increases in water‐use efficiency (WUE) due to reduced evapotranspirational water loss during CO_2_ capture (Borland et al., 2009; Cushman & Bohnert, 1999). This makes engineering an inducible CAM system into crops a potential tool for achieving sustainable global food security despite climate change‐induced more frequent droughts and high‐temperature events (Borland et al., 2009, 2014). The fact that CAM occurs in individual mesophyll cells of photosynthetic tissues, as opposed to the mesophyll/bundle sheath spatial separation required for C_4_ photosynthesis, and has evolved independently at least 66 times, supports the proposal that engineering CAM into C_3_ species to enhance their WUE should be achievable (Borland et al., 2014; Gilman et al., 2023).

During the repeated evolution of CAM from an ancestral C_3_ state, the circadian clock control of CAM‐associated metabolic steps was reprogrammed (Boxall et al., 2005; Hartwell, 2006). It has been hypothesised that this occurred through changes to signal transduction cascade(s) connecting the core circadian clock to CAM enzymes and metabolite transporters (Hartwell, 2006).

Previous work has established phosphoenolpyruvate carboxylase kinase (*PPCK1*) in the model CAM plant *Kalanchoë fedtschenkoi* (Hamet et Perrier) as a key circadian regulator of the pathway that synchronises and optimises primary nocturnal CO_2_ fixation by PPC in response to temporal signals from the core clock (Boxall et al., 2017; Carter et al., 1991; Hartwell et al., 1999; Nimmo et al., 1987). PPCK phosphorylates PPC in the dark period, reducing its sensitivity to feedback inhibition by malate (Nimmo et al., 1984). When *PPCK1* was silenced in transgenic *K. fedtschenkoi*, there was a 66% reduction in CAM‐associated nocturnal primary CO_2_ fixation and further regulatory disruption of the cycling of several core circadian clock genes, suggesting crosstalk between CAM and the core clock (Boxall et al., 2017). Similar evidence of crosstalk was reported for other transgenic RNAi lines of *Kalanchoë* lacking key CAM pathway enzymes, which resulted in the loss of CAM, with each line reverting to C_3_ (Boxall et al., 2020; Dever et al., 2015). However, the components of the signal transduction cascade that connects the core clock to *PPCK1*, or any other clock‐controlled CAM gene, remain unknown (Hartwell et al., 1996, 1999).

*In silico* analysis has led to the prediction of candidate circadian regulators of CAM in *K. fedtschenkoi*, and promoter regions of candidate CAM‐associated genes were enriched for known Arabidopsis clock‐controlled promoter motifs in the CAM crop *Ananas comosus* (pineapple) (Ming et al., 2015; Moseley et al., 2021). It is important to emphasise that the regulation of CAM is not limited to transcriptional control by the core‐clock. There is also extensive evidence for protein level control of CAM (Abraham et al., 2016, 2020; Guan et al., 2021; Nimmo et al., 1987; Schiller et al., 2025). However, there remains no functional, *in planta* research investigating the contribution of gene promoter regions and their *cis*‐elements to the circadian clock control of core CAM pathway genes. For example, given that PPCK1 is activated through *de novo* transcription and translation to deliver increased PPC phosphorylation each dark period during CAM, a logical hypothesis follows that the *PPCK1* promoter contains *cis*‐elements that are bound by transcription factors (TFs) that are themselves either part of the core circadian clock, or are part of the output pathway from the core circadian clock. The goal of this study was therefore to investigate the activity of the promoters of CAM‐associated clock‐controlled genes (CCCGs) in a novel diploid accession of *Kalanchoë laxiflora* (Cheng et al., 2026; Schiller et al., 2025) by generating stable transgenic lines expressing *PROMOTER::LUCIFERASE* reporter constructs. Our hypothesis was that the promoters of CCCGs would display high activity specifically in leaves performing CAM, and would be under robust circadian clock control with their activity peaking at the time of day that the CAM gene is known to function to optimise CAM.

Firefly luciferase (LUC)‐mediated bioluminescence has been widely adopted as a molecular‐genetic reporter for gene promoter activity in plants (Millar et al., 1992; Tindall et al., 2015). Early work using this tool coupled *LUC* to the promoter region of Arabidopsis *CHLOROPHYLL A/B BINDING PROTEIN2* (*AtCAB2*) and revealed this promoter to be under circadian clock control (Millar et al., 1992). Since that first seminal report, use of *AtCAB2::LUC* in circadian research has led to the discovery of many key components of the plant core clock including *TIMING OF CAB2 EXPRESSION1* (*TOC1*), *LUX ARRYTHMO* (*LUX*), *EARLY BIRD* (*EBI*) and *TIME FOR COFFEE* (*TIC*) (Ashelford et al., 2011; Ding et al., 2007; Hazen et al., 2005; Millar et al., 1995; Strayer et al., 2000). In addition, a number of core‐clock gene promoters have been shown to drive circadian rhythms of *LUC* in stable transgenic lines, including *TOC1*, *CIRCADIAN CLOCK ASSOCIATED 1* (*CCA1*), *LATE ELONGATED HYPOCOTYL* (*LHY*), *GIGANTEA* (*GI*), *EARLY FLOWERING3* (*ELF3*), *ELF4* and several *PSEUDO RESPONSE REGULATOR* (*PRR*) genes (Doyle et al., 2002; Greenwood et al., 2019; Locke et al., 2006; Nakamichi et al., 2004; Nimmo & Laird, 2021; Palagyi et al., 2010; Salome & McClung, 2005). Despite the *CAB2* gene being a keystone for understanding the molecular‐genetics of the plant clock, its promoter region has not been examined in a CAM species, and use of *PROMOTER::LUC* reporters to study circadian biology in plant species other than Arabidopsis has remained remarkably limited.

For the current work, putative promoter regions of CCCGs *K. laxiflora GLUCOSE 6‐PHOSPHATE:PHOSPHATE TRANSLOCATOR2* (*KlGPT2*) and *KlPPCK1*, and the non‐CAM clock‐controlled gene *KlCAB2*, were cloned upstream of the *LUC+* reporter and used to generate stable transgenic lines in *K. laxiflora* and C_3_ Arabidopsis. The other CCCG promoter studied here was that of *GPT2* (Kammerer et al., 1998). During CAM, GPT may function both for nocturnal export of glucose 6‐phosphate (G6P) from chloroplasts to support phosphoenolpyruvate (PEP) provision for primary CO_2_ fixation, and also for the uptake of G6P into chloroplasts in the light period to feed starch regeneration from the pyruvate generated by malate decarboxylation (Hausler et al., 2000). In the facultative CAM species *Mesembryanthemum crystallinum* (common iceplant), McGPT activity and *McGPT2* promoter activity and transcript abundance all increased co‐incident with CAM‐induction by salt‐ or drought‐stress (Azad et al., 2013; Cushman et al., 2008; Hausler et al., 2000; Kore‐eda et al., 2005). *McGPT2* transcript levels displayed robust circadian oscillations under free‐running constant light and temperature (LL) conditions in plants induced into CAM (Cushman et al., 2008; Kore‐eda et al., 2005). In wild‐type *K. laxiflora*, transcripts of *KlGPT2* oscillated robustly under LL and were down‐regulated when CAM‐associated *PPC1* was silenced using RNAi, which led to *K. laxiflora* reverting to C_3_ (Boxall et al., 2020).

**Figure 1:**
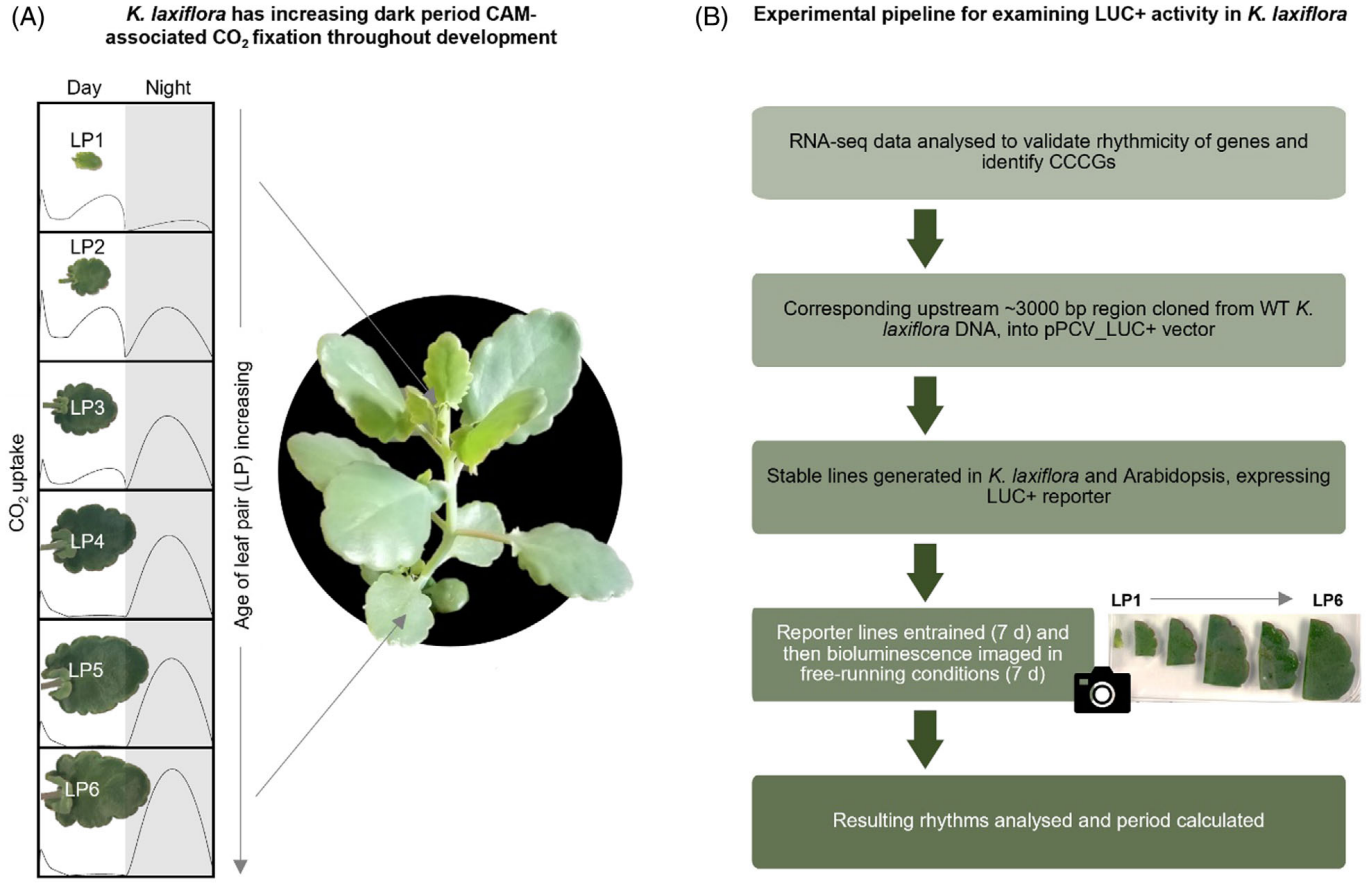
Overview of the *Kalanchoë laxiflora* diploid model system and pipeline for *K. laxiflora* gene *PROMOTER::LUC+* reporter experiments. (A) *Kalanchoë laxiflora* leaf pairs develop in a sequential manner from the shoot apical meristem down the stem. There is a corresponding gradient of increasing Crassulacean acid metabolism (CAM)‐associated dark period CO_2_ fixation that peaks in leaf pair 6 (LP6), where all atmospheric CO_2_ assimilation occurs during the dark period, and leaves perform full CAM. Beyond LP6 full CAM continues (gas exchange plots adapted from Figure S4; Boxall et al., 2020). (B) Pipeline and experimental set‐up for *in planta PROMOTER::LUC+* experiments using LP1–LP6 of *K. laxiflora* or Arabidopsis to investigate circadian clock control of *K. laxiflora* promoters across the C_3_‐CAM gradient, or in a C_3_ species.

*Kalanchoë* species are currently the main model systems for functional genomics studies of CAM, especially *K. fedtschenkoi* and *K. laxiflora* (Boxall et al., 2017, 2020; Ceusters et al., 2021; Dever et al., 2015; Hurtado‐Castano et al., 2023; Lefoulon et al., 2020). These species have publicly available genomes and transcriptomes, and stable transgenic lines can be generated via tissue culture using *Agrobacterium tumefaciens* (Cheng et al., 2026; Hartwell et al., 2016; Meng et al., 2026; Wang et al., 2019; Yang et al., 2017). A diploid accession of *K. laxiflora* that can set viable seed has recently been identified, further enhancing the amenability of this system as a molecular‐genetic model for CAM and other novel adaptations of plant biology found in the genus *Kalanchoë* (Cheng et al., 2026; Hartwell et al., 2016; Schiller et al., 2025). Here, this diploid accession of *K. laxiflora* was used to establish a robust, *in planta* LUC imaging system for studying CCCG promoter activity. The generated transgenic lines were used to investigate links between the core‐clock and the regulation of the promoter regions of CCCGs. *Kalanchoë laxiflora* provides a powerful system for studying the regulation of CCCGs as the youngest leaves (leaf pair 1; LP1) perform ‘C_3_‐like’ CO_2_ fixation, where all net atmospheric CO_2_ assimilation occurs in the light period, whereas mature LP6 perform strong CAM, with the majority of 24‐h atmospheric CO_2_ fixation occurring in the dark period (Figure 1) (Boxall et al., 2020). *KlCAB2p::LUC+* expressed in *K. laxiflora* provided a positive control that established the imaging system and demonstrated robust circadian oscillations of LUC+ activity, and lines expressing *KlGPT2p::LUC+* revealed that the activity of this CAM gene promoter was under circadian clock control. Comparison of circadian rhythms driven by *KlCAB2p* and *KlGPT2p* suggested that *KlGPT2p* activity was coupled with CAM development as leaves matured, whereas *KlCAB2p* activity displayed its highest amplitude oscillations in the youngest leaf pairs. In addition, the circadian period of *KlCAB2p::LUC+* changed with leaf development. Unexpectedly, the promoter of the classic CCCG *KlPPCK1* was unable to drive any detectable LUC+ activity in multiple independent *K. laxiflora* transgenic lines.

To test whether the circadian clock output signalling pathways that control the *K. laxiflora* promoters are conserved in a C_3_ species, each *Klp::LUC+* construct was also transformed into Arabidopsis. The timing of the peak of *KlCAB2p::LUC+* activity shifted by up to 12‐h in Arabidopsis compared to the peak timing in *K. laxiflora* CAM leaves. Overall, the results identified differences in the timing of signalling from the core‐clock in C_3_ Arabidopsis as compared to the CAM leaves of *K. laxiflora*, but there is clear conservation of the clock‐controlled promoter motifs in the *Kalanchoë* promoter due to its circadian activity in Arabidopsis.

## RESULTS

**Figure 2:**
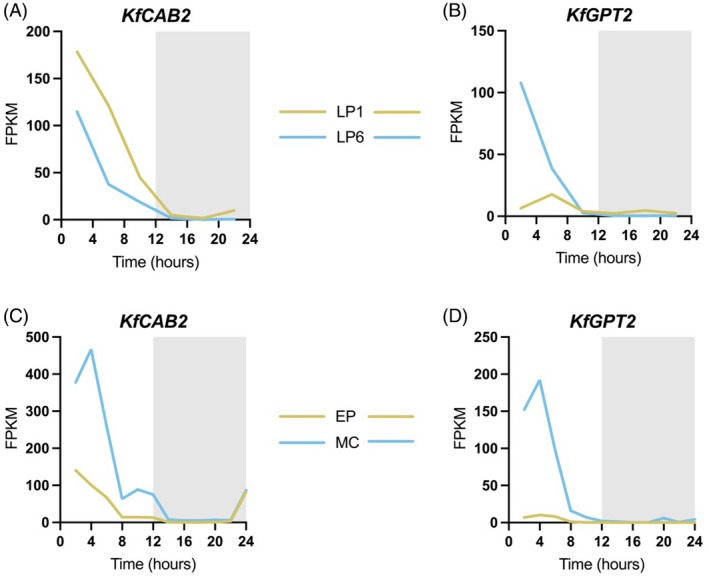
*KfCAB2* and *KfGPT2* transcripts oscillated in abundance over a 12‐h‐light/12‐h‐dark cycle in both different leaf ages and separated tissue types from mature Crassulacean acid metabolism (CAM) leaves of *Kalanchoë fedtschenkoi*. (A–D) Rhythmic variation over the light/dark cycle of the transcript abundance of *KfCAB2* and *KfGPT2*. (A, B) Light/dark transcript oscillations for whole LP1 and LP6 that were sampled every 4‐h. (C, D) Epidermal peels were separated from underlying mesophyll cells using full CAM leaves (pooling samples from LP6, 7 and 8), with sampling time points every 2‐h. *Y*‐axis values display the fragments per kilobase of transcript per million mapped reads (FPKM) values generated through RNA‐seq analysis. Transcript abundances from CAM‐performing mesophyll cells (MC) and leaf pair 6 (LP6) are in blue, whereas samples from epidermal peels (EP) and C_3_‐performing leaf pair 1 (LP1) are in yellow. Samples were collected from wild‐type *K. fedtschenkoi* leaves pre‐entrained under 12‐h‐light/12‐h‐dark cycles.

Data from existing RNA‐seq datasets for *K. fedtschenkoi* demonstrated that the transcript abundance of *CAB2* and *GPT2* oscillated robustly over a 12‐h‐light/12‐h‐dark cycle within both whole CAM leaves (leaf pair 6; LP6) and separated mesophyll tissue from CAM leaves (Figure 2). *Kalanchoë GPT2* transcripts oscillated with highest amplitude in CAM performing LP6, and in leaf mesophyll tissue separated from full CAM leaves, LP6, 7 and 8 (Figure 2B,D).

### *KlCAB2p* drove robust rhythms of LUC+ activity in *K. laxiflora*

**Figure 3:**
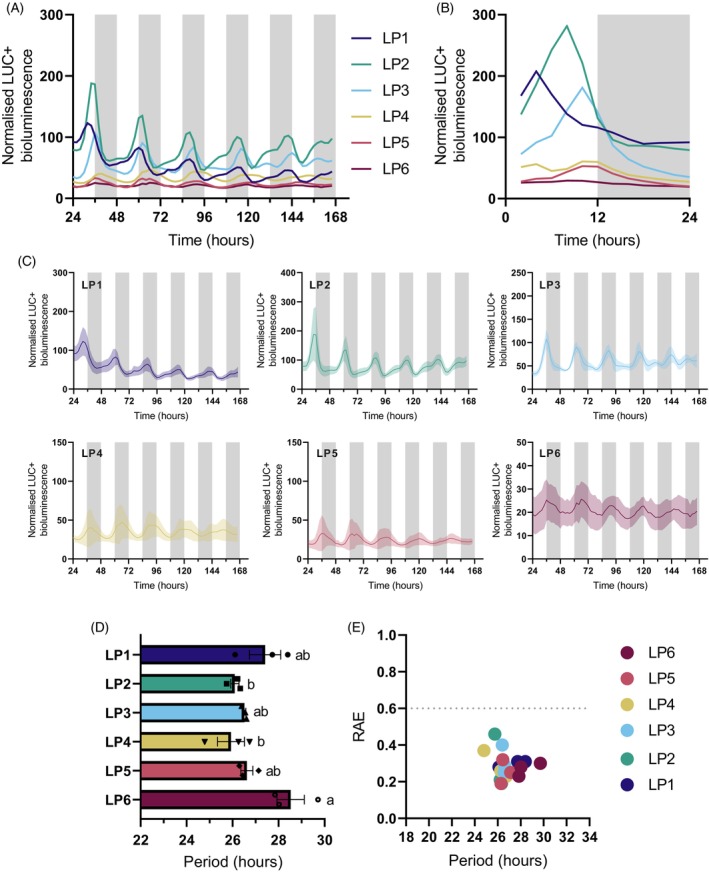
*KlCAB2p::LUC+* drove robust circadian rhythms of luciferase activity in a leaf age dependent manner in *Kalanchoë laxiflora*. (A) Mean LUC+ expression from for all *K. laxiflora* leaf pairs from ZT24 to ZT166. (B) Bioluminescence for ZT0–ZT24 in LL for *K. laxiflora*. (C) From ZT24 to ZT166 of *KlCAB2p::LUC+* activity for each individual leaf pair. The bold line represents the mean, and the shaded area is SEM. The *y*‐axis scales vary. (D, E) Period and relative amplitude error (RAE) calculated from ZT24 to ZT96 h using fast Fourier transform‐non‐linear least squares (FFT‐NLS). Three biological replicates of *K. laxiflora* diploid expressing *KlCAB2p::LUC+* were imaged under LL for 7 days at 15°C. White and grey bars on graphs represent the subjective light and dark periods, respectively. Error bars represent SEM. Only replicates that were significantly rhythmic when tested using Biodare2 eJTK and BD2 Classic with a *P* value <0.005 after Benjamin–Hochberg correction were included in period. An ordinary one‐way anova with Tukey's multiple comparisons was used to assess differences in period, where *P* = 0.0158. The lowercase letters ‘a’, ‘ab’ and ‘b’ indicate whether or not two period values were significantly different from one another. The period lengths that share the same letter(s) were not significantly different from one another.

Stable *K. laxiflora* transgenic lines carrying the *KlCAB2p::LUC+* construct exhibited robust circadian rhythms of bioluminescence across all LPs (Figure 3; Table S1). Reassuringly, the results mirrored previously published results for *AtCAB2p::LUC* in Arabidopsis in terms of their robustness and consistency (Millar et al., 1992). The rhythm robustness of these free‐running rhythms was supported by statistical testing for circadian rhythmicity using Biodare2 (Moore et al., 2014; Zielinski et al., 2014). The characteristics of the free‐running rhythms of *KlCAB2p::LUC+* varied with leaf age.

In LP1, peak *KlCAB2p::LUC*+ activity occurred earlier in the subjective light, peaking at ZT04, whereas in older LPs the LUC+ luminescence peaked later in the light period (Figure 3). LP2 had the highest relative bioluminescence, whereas older leaves that perform full CAM displayed lower amplitude LUC+ oscillations (Figure 3A–C). The period length of the free‐running circadian rhythms was longer for LP6 than for LP2 and LP4 (Figure 3D). Furthermore, the range of periods measured for this construct (~26–28‐h) was markedly longer than those of ~21‐h reported previously for the CAM‐associated CO_2_ fixation rhythm under LL conditions in LP6, which were conducted at the same temperature (Boxall et al., 2020). In addition, the circadian rhythm of CAM‐associated CO_2_ fixation under LL at 15°C in the OBG diploid accession of *K. laxiflora* used here also had a shorter free‐running period (~22.5–25‐h) (Figure S2) than that for *KlCAB2p::LUC+* (~26–28‐h) (Figure 3).

### *KlGPT2p* drove robust LUC+ rhythms that peaked in amplitude in LP3, but *KlPPCK1p* did not generate detectable LUC+

**Figure 4:**
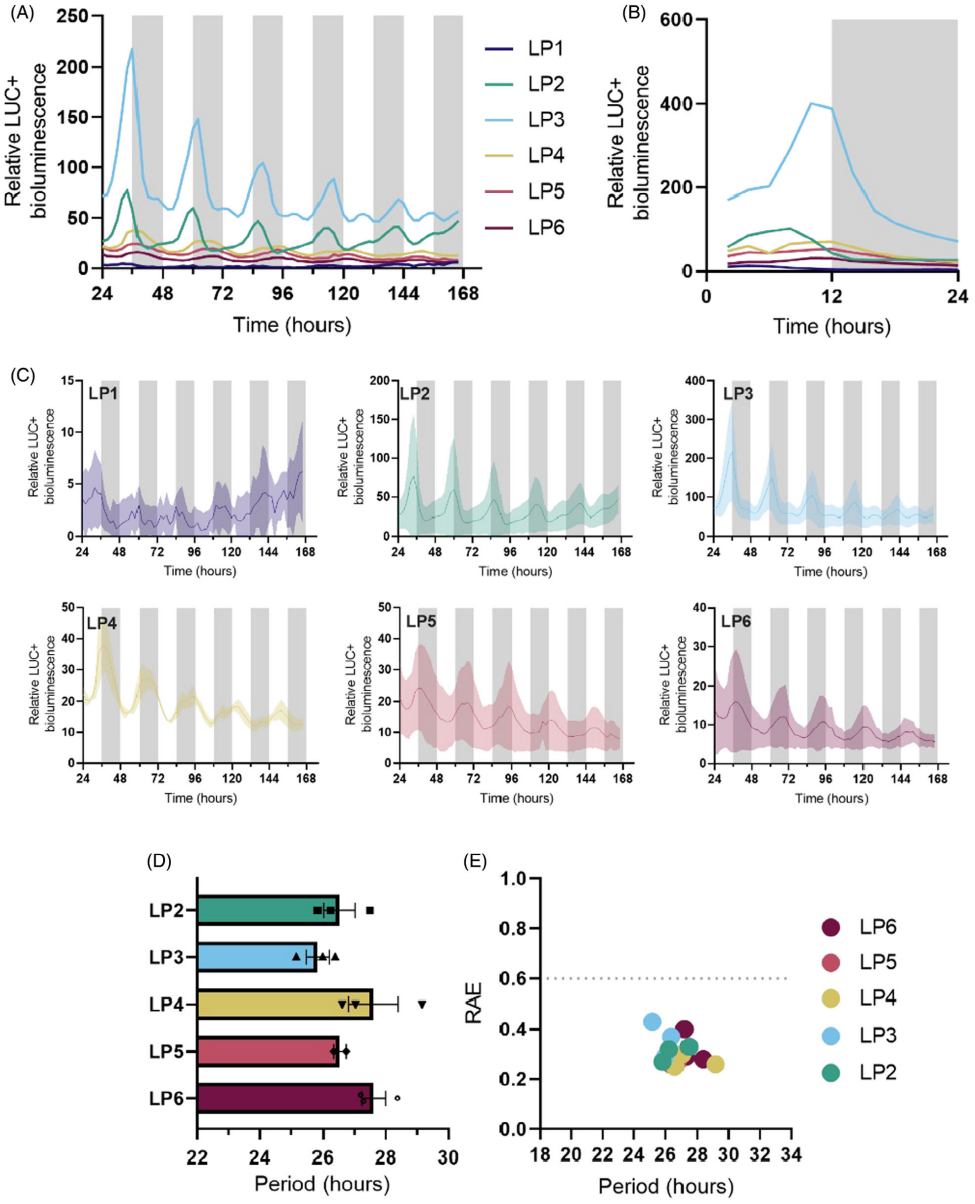
*KlGPT2p* drove robust circadian rhythms in *Kalanchoë laxiflora*. (A) Mean LUC+ activity for each measured *K. laxiflora* leaf pair from ZT24 to ZT166. (B) Bioluminescence for ZT0–ZT24 under LL for *K. laxiflora*. (C) *KlGPT2p::LUC+* expression for each individual leaf pair from ZT24 to ZT166. The bold line represents the mean, and the shaded area is the SEM. (D, E) Period and relative amplitude error (RAE) calculated from ZT24 to ZT96 using fast Fourier transform‐non‐linear least squares (FFT‐NLS). Three biological replicates of a stable transgenic line of *K. laxiflora* OBG diploid expressing *KlGPT2p::LUC+* were imaged under LL conditions at 15°C for 7 days. White and grey bars on graphs represent the subjective light and dark periods, respectively. Error bars represent SEM. Only replicates that were significantly rhythmic when tested using Biodare2 eJTK and BD2 Classic with a *P* value <0.005 after Benjamin–Hochberg correction were included in period. An ordinary one‐way anova showed no significant differences in period values across leaf pairs, where *P* = 0.8591 (*P* > 0.05).

*KlGPT2* transcripts oscillated with greater amplitude in older *K. laxiflora* leaves that perform full CAM (Figure 2B,D) (Boxall et al., 2020). In contrast to the rhythms of *KlCAB2p*, for which rhythm amplitude varied by small increments with leaf age (Figure 3), the mean activity of *KlGPT2p::LUC+* peaked strongly in LP3 relative to all other LPs (Figure S6). The bioluminescence signal was around 3.5‐fold higher than the next highest level detected in LP2 (Figure 4; Figure S6). Analysis also showed that the LUC+ signal measured for LP1 was not rhythmic, with a high relative amplitude error above 0.6. The data also failed to pass the statistical threshold for rhythmicity tests with a Benjamin–Hochberg corrected *P* value >0.005 (Figure 4C; Table S2). All other leaf ages were rhythmic in at least one replicate based on these tests (Figure 4E; Table S2). Across LPs, *KlGPT2p::LUC+* activity peaked towards subjective dusk (Figure 4A–C), revealing that the timing of the peak promoter activity was shifted relative to the timing of the peak steady‐state transcript level at the start of the light period under LD cycles (Figure 2) (Boxall et al., 2020). Free‐running period lengths were more consistent across leaf ages than those measured for *KlCAB2p*, although they were of a similar length overall at between 26‐ and 28‐h (Figure 4D).

Intriguingly, *KlPPCK1p::LUC+* did not generate a detectable level of luminescence in *K. laxiflora* leaves of any age, with minimal luminescence being detected across the full 7‐days for numerous independent transgenic lines (Figure S1).

### *KlCAB2p::LUC+* drove robust circadian rhythms in Arabidopsis with a phase shift in the timing of peak LUC+ activity relative to CAM‐performing LP6 of *K. laxiflora*

The *KlCAB2p::LUC+*, *KlGPT2p*::*LUC+* and *KlPPCK1p::LUC+* constructs were transformed into Arabidopsis to investigate whether or not the output signal transduction pathway from the core circadian clock in this C_3_ species could control these *K. laxiflora* gene promoters. Two key questions were explored. Firstly, could the *K. laxiflora* promoters be driven by the core‐clock in C_3_ Arabidopsis? Secondly, if the *K. laxiflora* promoters were under clock control in C_3_ Arabidopsis, was the period length and phasing the same or different relative to the regulation in *K. laxiflora*?

**Figure 5:**
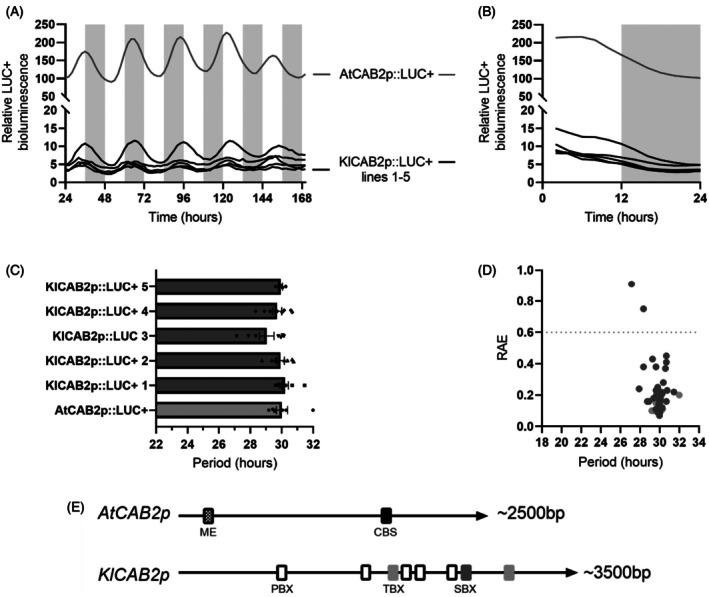
*KlCAB2p::LUC+* drove robust circadian oscillations in Arabidopsis with similar phase and period to those driven by *AtCAB2::LUC+*. (A) Mean LUC+ expression from ZT24 to ZT166. (B) Mean LUC+ expression from ZT0 to ZT24. (C, D) Period and relative amplitude error (RAE) calculated from ZT24 to ZT96 with fast Fourier transform‐non‐linear least squares (FFT‐NLS). Eight wells containing ~10 Arabidopsis seedlings expressing *AtCAB2p::LUC+* and five independent lines expressing *KlCAB2p::LUC+* were imaged under LL for 7 days at 15°C. White and grey bars on graphs represent the subjective light and dark periods, respectively. Error bars represent SEM. Only replicates that were significantly rhythmic when tested using Biodare2 eJTK and BD2 Classic with a *P* value <0.005 after Benjamin–Hochberg correction were included in period calculations. An ordinary one‐way anova found no differences in period. (E) Circadian promoter motifs mapped onto putative promoters for *AtCAB2* and *KlCAB2*: the Morning Element (ME: AACCAC), *CIRCADIAN CLOCK ASSOCIATED1*‐binding site (CBS: AAAAAATCT), protein box (PBX: ATGGGCC), telo‐box (TBX: AAACCCT) and starch box (SBX: AAGCCC).

**Figure 6:**
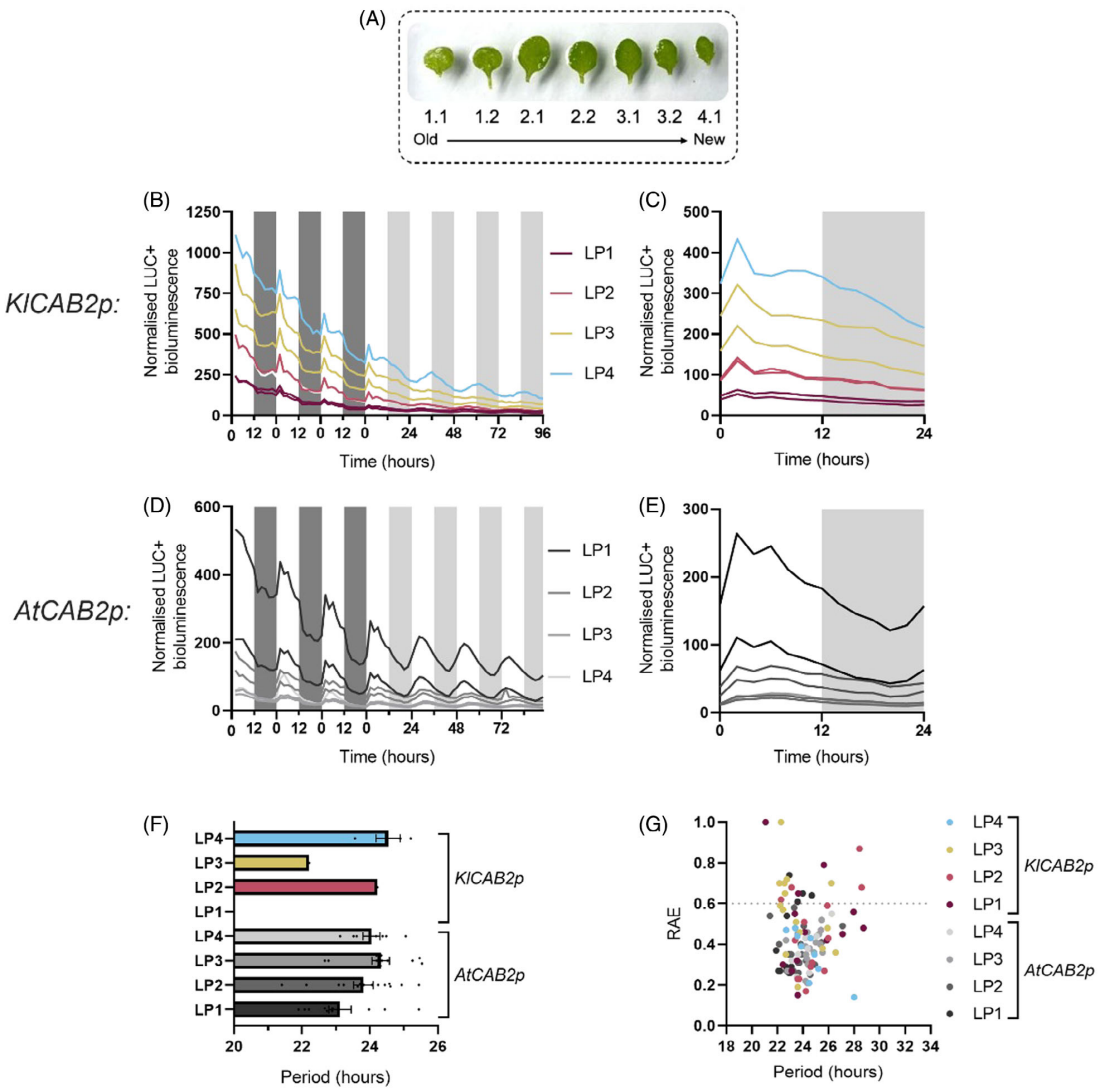
*KlCAB2p* had higher activity in the youngest leaves of Arabidopsis, whereas *AtCAB2p::LUC+* activity was highest in the oldest leaves. (A) Nine replicates of a 21‐day‐old stable transgenic line of Arabidopsis expressing *KlCAB2p::LUC+* or *AtCAB2p::LUC+* were imaged in LD for 3‐days and released into LL and measured for a further 4‐days at 15°C. (B, D) Mean LUC+ bioluminescence for each leaf number for the entire imaging period plotted in ZT. (C, E) LUC+ bioluminescence from ZT0 to ZT24. (F, G) Period and relative amplitude error (RAE) calculated in LL using data from ZT24 to ZT96 analysed with fast Fourier transform‐non‐linear least squares (FFT‐NLS) with linear detrending. Dark grey bars on the graphs are true dark, while light grey bars represent subjective dark. Leaves were numbered according to developmental appearance. Error bars represent SEM. Periods are only shown for replicates that were significantly rhythmic when tested using Biodare2 eJTK and BD2 Classic with a *P* value <0.005 after Benjamin–Hochberg correction. An ordinary one‐way anova found no differences in period.

The *KlCAB2* promoter drove free‐running rhythms of LUC+ activity in both 7‐day‐old Arabidopsis seedlings and mature leaves of 21‐day‐old plants, whereas the CAM‐associated *KlGPT2p* did not generate a detectable level of LUC+ bioluminescence (Figures 5 and 6). *KlCAB2p* drove relatively low levels of LUC+ expression in comparison to the native *AtCAB2p* (Figure 5A,B). Given this large difference in the bioluminescence intensity detected for *KlCABp* relative to the much brighter *AtCAB2p*, experiments were carried out to check that the measured bioluminescence signal was not due to spill‐over light signal from neighbouring *AtCAB2p::LUC+* seedlings that were under the imaging camera along with the *KlCAB2p::LUC+* seedlings. This possibility was ruled out because robust circadian rhythms of *KlCAB2p::LUC+* in Arabidopsis were detected when there were no *AtCAB2p::LUC+* seedlings on the imaging plate placed under the CCD‐camera (Figure S4).

This check for spill‐over between the different genetic lines was important because the two promoters from different species drove rhythms with very similar phasing when expressed in Arabidopsis, with a characteristic peak at the start of the light period (Figure 5B). The same result was also observed in experiments using older Arabidopsis leaves (Figure 6C,E). This contrasted with the phasing of *KlCAB2p::LUC+* in *K. laxiflora*, which only peaked at the start of the subjective light in the youngest leaves that use C_3_ photosynthesis (Figure 3B). It was particularly intriguing that the phasing and period of both *KlCAB2p* and *AtCAB2*p activity in Arabidopsis were similar despite the two promoters sharing very limited sequence similarity or conserved islands of known circadian clock‐regulated promoter motifs (Figure 5E).

To investigate whether the trend of varying period length with leaf age measured for *KlCAB2p::LUC+* in *K. laxiflora* leaves (Figure 3D) was also a feature of the regulation of this reporter construct in Arabidopsis, imaging was performed on individual leaves from 21‐day‐old Arabidopsis rosettes expressing *KlCAB2p::LUC+* or *AtCAB2p::LUC+* (Figure 6). Leaves were numbered according to their order of developmental appearance, such that the first pair of true leaves to appear after the cotyledons were the oldest leaves, numbered 1.1 and 1.2, and so on (Figure 6A) (Kim et al., 2016). From ZT0 to ZT24, *KlCAB2p::LUC+* activity in Arabidopsis peaked shortly after subjective dawn (Figure 6C), consistent with the results for 7‐day‐old plants, and supporting the conclusion that imaging detached leaves did not fundamentally change the circadian activity of the reporter (cf. Figures 5B and 6E). As was the case for individual replicates of *K. laxiflora* leaves, Arabidopsis leaves were not included in the analysis if they did not pass the statistical tests for rhythmicity. Rhythm robustness for *KlCAB2p::LUC+* activity was reduced in detached Arabidopsis leaves compared to whole seedlings (cf. Figures 5D and 6G). Younger LPs expressing *KlCAB2p* were more commonly arrhythmic (Figure 6G). However, the youngest leaves (LP4) did generate robust rhythms for all replicates (Figure 6G).

All LPs expressing *AtCAB2p::LUC+* were rhythmic and the activity peaked in the oldest leaves (LP1) rather than the youngest (LP4), the opposite of the result for *KlCAB2p* (Figure 6). This inversion in terms of the age of the leaves in which each promoter drove higher activity of LUC+ was complete across leaf development (Figure 6B,D). Secondly, the range of period values for rhythmic leaves was similar for *KlCAB2p* and *AtCAB2p*, varying around 24‐h (Figure 6F,G). The general trend for the leaves expressing *AtCAB2p::LUC+* showed that period shortened as leaves aged, but this trend was only qualitative, as only LP1 and LP3 had a statistically significant difference in period length.

## DISCUSSION

This study investigated the molecular‐genetic components that underpin the daily temporal optimisation of CAM by the circadian clock using the model species *K. laxiflora*. Stable transgenic lines were established that expressed firefly LUC under the control of candidate clock‐controlled promoter regions. The observed robust circadian rhythms of LUC+ bioluminescence driven by *KlCAB2p* across all LPs in *K. laxiflora* likely reflect the fundamental role of the *CAB2* gene in supporting the light reactions of photosynthesis in all plants, irrespective of CAM activity. The free‐running period lengths for the *KlCAB2p::LUC+* reporter in *K. laxiflora* leaves were longer than the ~22.5–25‐h period of the rhythm of CO_2_ gas exchange measured for LP6 of wild type plants of the same *K. laxiflora* accession (Figure S2). *KlCAB2p* in *K. laxiflora* had a period ranging from 26.1‐ to 28.52‐h, depending on the LP (Figure 3D). Period lengths were similar for *KlGPT2p* (26.52‐ to 27.78‐h; Figure 4D), indicating long free‐running periods were not specific to *KlCAB2p*. A free‐running circadian period of around 24‐h is typical for most organisms, including the CAM‐associated CO_2_ assimilation circadian rhythm of *K. laxiflora* OBG (Figure S2). One explanation for the differences observed here may be temperature compensation resulting from the cooler conditions in which plants were imaged. However, these conditions were chosen to replicate the LL conditions used both for previously published LL CO_2_ gas exchange circadian rhythms measured for detached leaves of *K. laxiflora* tetraploid, which maintained a period around 21–22 h (Boxall et al., 2020), and for the LL CO_2_ exchange data for *K. laxiflora* OBG diploid presented here (Figure S2). The reasons for this difference in free‐running period length between promoter activity and gas exchange rhythms remain to be determined, but this clear difference in period length for different circadian clock outputs could reveal novel layers of regulation that fine‐tune timing as temporal signals are passed from the core clock and sequentially cascade all the way to the timing of the whole leaf gas exchange physiology.

Interestingly, the circadian period of *KlCAB2p::LUC+* in *K. laxiflora* was significantly longer for LP6 than for LP2 or LP4 (Figure 3D), which contributed to a shift in phase across LPs, with the peak activity phased later in older leaves (Figure 3B; Figure S6). Differences in period length for LP1, which had on average a longer period than the other young LPs (Figure 3D), could be linked to the fact that this youngest pair of leaves was still undergoing initial development and progressing to attaining full photosynthetic competence; it was noteworthy in this context that LP1 is noticeably paler green than the other leaf pairs (Figure 1A). For the other LPs, the initiation of CAM coincident with progressive leaf maturation, all the way to them becoming fully developed, sink leaves that use full CAM, could contribute to the changes in period length measured here. It has been shown in other CAM species that the C_3_‐CAM transition induced by stress is accompanied by a large transcriptomic shift (Cushman et al., 2008). Longer circadian periods in older leaves may also be related to the developmental transition of the leaves as they age.

Plasticity of the clock during ageing has been widely reported in mammalian systems, with period shortening with age in both rats and humans, while in insects, circadian period lengthened with increasing age (Duffy & Czeisler, 2002; Giebultowicz & Long, 2015; Witting et al., 1994). In plants, circadian period was ~1‐h shorter in older leaves relative to younger leaves when calculated for plants of the same age (Kim et al., 2016). Leaves from plants of increasing age have also been examined, which revealed a shortening of the *AtCCA1p::LUC* free‐running period from 25.7‐h at 14‐days to 23.9‐h at 35‐days (Jung et al., 2024). This was related to phasing alterations of many core‐clock components and a transcriptomic reprogramming of timing of gene transcript abundance rhythms as the plants aged. A key distinction was that genes that peaked in the light shortened their period throughout ageing, whereas the reverse was the case for dark period phased genes (Jung et al., 2024). In the crops *Triticum aestivum* (wheat) and oilseed rape (*Brassica napus*), measurements of the circadian rhythm of delayed fluorescence of leaves revealed that period length changed during ageing for both whole plants and individual leaves of different ages (Rees et al., 2019). The findings led to the conclusion that the clock was running faster in young plants before senescence began (Kim & Hong, 2019; Rees et al., 2019). The transition to CAM as leaves develop in *K. laxiflora* is accompanied by increased nocturnal CO_2_ fixation and malate accumulation associated with CAM, and accompanying rewiring of the transcriptomic regulatory landscape (Boxall et al., 2020; Hartwell et al., 1999; Jones, 1975). Leaves also transition from acting as sink leaves to source leaves as they develop and mature, which could contribute to the measured shifts in period length. Future studies should investigate the regulation of the promoters of core‐clock genes in *Kalanchoë*, such as *TOC1*, *CCA1* or *GI*, as well as a wider diversity of CCCGs, across the leaf developmental gradient in order to untangle the relationship between leaf ageing and the circadian control of CAM.

*KlGPT2p* drove robust rhythms of LUC+ bioluminescence in all LPs except LP1, and this result was observed consistently in multiple, independent, stable transgenic lines of *K. laxiflora* (Figure 4A,C). A key difference in the temporal expression patterns of LUC+ driven by the *KlGPT2p* compared to *KlCAB2p* in *K. laxiflora* was the brighter peak of LUC+ signal driven by *KlGPT2p* in LP3, which was 3.5‐fold higher than the next brightest in LP2 (Figure S6). These results revealed a strong peak of promoter activity in LP3, coincident with CAM‐associated nocturnal CO_2_ assimilation contributing the majority of 24‐h CO_2_ assimilation (Figure 1A). Taken with previous findings that the high abundance and light/dark cycling of *KlGPT2* transcript abundance was dependent on a functional CAM cycle, these findings support the conclusion that *GPT2p* is activated as leaves grow and mature as part of the developmental induction of CAM in *Kalanchoë* (Boxall et al., 2020). *GPT2* may play a reduced role in active CAM in LP4, 5 and 6, wherein ‘full‐CAM’ occurs, with almost all atmospheric CO_2_ fixation occurring in the dark. Alternatively, the functional GPT2 protein may remain stable after it is installed in the chloroplast envelope following the peak of transcript abundance in LP3. Further investigations into the KlGPT2 protein abundance and stability across all LPs, and its transport activity in isolated chloroplasts, would be required to understand the balance between changes in promoter activity across leaf ages and the abundance and activity of the encoded protein.

A commonality between the *KlCAB2p* and *KlGPT2p* activities was the waves in intensity of bioluminescence of LUC+ across the leaves, observed in time‐lapse videos (Figure S3). This observation provided further evidence that the core‐clock is a complex integration of single‐cell autonomous clocks coupled between neighbouring cells, resulting in waves of peak gene activity as cells communicate (Beck et al., 2001; Gould et al., 2009, 2018; Rascher et al., 2001).

The *KlPPCKp::LUC+* construct used here to generate stable transgenic lines of *K. laxiflora* OBG diploid consisted of ~2500‐bp upstream from the *KlPPCK1* ATG start codon. This candidate promoter region was not able to drive detectable LUC+ bioluminescence in multiple, independent transgenic lines of *K. laxiflora* or Arabidopsis. The lack of LUC+ bioluminescence for *K. laxiflora* lines carrying the *KlPPCK1p::LUC+* reporter may indicate that the cloned ~2500 bp putative promoter region does not possess all of the necessary regulatory sequence motifs required for the promoter to drive even low levels of the *LUC*+ reporter. Either key promoter motifs are present further than ~2500‐bp upstream, or there may be distal promoter enhancer elements at large distances from the *PPCK1* transcribed region. The possibility that key promoter motifs are located further upstream than ~2500 bp is supported by studying the chromosomal context of the CAM‐associated *PPCK1* in several telomere‐to‐telomere *Kalanchoë* genomes that have been published recently (Meng et al., 2026). In these high‐quality genomes for *K. fedtschenkoi*, *K. daigremontiana* and *K. marnieriana*, which are all in the same leaf plantlet‐forming ‘Bryophyllum’ sub‐clade of the genus *Kalanchoë* that *K. laxiflora* belongs to (Rodewald et al., 2025), the CAM *PPCK1* gene is found in the middle of chromosome 11. Looking back along the chromosome from the 5′‐end of the *PPCK1* open reading frame reveals that the intergenic region between *PPCK1* and the next annotated gene is between ~15 000 and ~17 000 bp long. The upstream gene is annotated on the antisense strand pointing in the opposite direction to *PPCK1*. Thus, the promoter responsible for receiving time‐of‐day signals from the core circadian clock and driving high activity of PPCK1 in mesophyll cells in the dark period in leaves performing CAM may well stretch for many thousands of base pairs upstream beyond the ~2500 bp fragment cloned and tested here. Given the importance of PPCK1 for the optimised circadian control of CAM, and the critical need to understand how PPCK1 transcript levels are increased each night in response to the circadian clock to produce the active protein kinase, further work must explore longer upstream candidate promoter regions as well as including sequences spanning the start of the coding region of the *PPCK1* gene in *Kalanchoë*.

In *M. crystallinum*, a stress responsive bZIP TF, *ELONGATED HYPOCOTYL5* (*McHY5*), was found to activate an *McPPCK1* promoter LUC reporter when co‐infiltrated with the reporter such that both were expressed transiently in leaves of C_3_ *Nicotiana benthamiana* (Perron et al., 2025). By contrast, AtHY5 could not activate the *McPPCK1* promoter when co‐infiltrated into *N. benthamiana*. These results led the authors to conclude that McHY5 may have been co‐opted to perform a specific role in regulation of the CAM‐specific *PPCK1* in *M. crystallinum* (Perron et al., 2025). However, given that the study only demonstrated activation of the *McPPCK1* promoter by McHY5 protein through co‐infiltration of the constructs into heterologous, C_3_ *N. benthamiana* leaves, and the result relied on constitutive expression of McHY5 with the reporter construct, it remains to be seen whether McHY5 binds to and activates the *McPPCK1* promoter to drive the observed *in planta* peak of McPPCK1 activity in the dark period in CAM‐induced *M. crystallinum*. These intriguing preliminary results for the *McPPCK1* promoter do, however, suggest that it would be interesting to test whether the *Kalanchoë* HY5 protein binds to and activates the *KlPPCK1* promoter in *K. laxiflora* as part of future work.

### *KlCAB2p* had altered timing of peak activity when expressed in Arabidopsis compared to its regulation in its native *K. laxiflora*

Introduction of the *K. laxiflora p::LUC+* constructs into Arabidopsis allowed for comparative analysis of their regulation in a C_3_ species, which provided a method to explore whether or not the circadian output pathway that controls the promoters in *K. laxiflora* is conserved in Arabidopsis. The findings revealed differences between the activity and timing of *KlCAB2p* in the two species, emphasising the need to study circadian molecular genetics and signalling pathways in the native environment of the promoter of interest. In the period from ZT0 to ZT24 for the experiment with 7‐day‐old Arabidopsis seedlings, *KlCAB2p* peaked at subjective dawn (Figure 5B). By contrast, *KlCAB2p* in *K. laxiflora* peaked later at subjective dusk in most leaf pairs (Figure 3B). This species‐dependent temporal shift in the timing of activity of *KlCAB2p* demonstrated that the *K. laxiflora* promoter most likely coupled to the Arabidopsis core clock in the same way that the *AtCAB2p* does given that it peaked at the same time as *AtCAB2p*.

To further explore the regulation of the activity of the *KlCAB2p* in Arabidopsis, single leaves of different developmental ages were sampled from mature plants expressing either *KlCAB2p* or *AtCAB2p*. By comparison to the experiments using 7‐day‐old seedlings (Figure 5), it was confirmed that rhythmicity was not lost by detaching leaves of plants expressing *AtCAB2p::LUC+*, but there were fewer persistent rhythms detected for the leaves from plants expressing *KlCAB2p* (Figure 6). Despite this, both versions of the promoter had similar phasing with peak activity in the early subjective light period, just as they did in young Arabidopsis seedlings, and again the timing was similar for both promoters. This shared phasing between *KlCAB2p and AtCAB2p* cannot be explained by conserved circadian motifs shared by the two promoter sequences, as *AtCAB2p* had a CCA1‐binding site (AAAAAATCT) and a Morning Element (ME; CCACAC) that were both absent as exact sequence matches within *KlCAB2p* (Figure 5E). There were however several ME‐like (CCACA) elements throughout the ~3000 bp *KlCAB2p* sequence (Hsu et al., 2013). In addition, other promoter motifs linked with circadian control of genes in Arabidopsis were present in the *KlCAB2* promoter sequence. It was noteworthy in this context that the *KlCAB2p* sequence included four copies of the protein box (PBX: ATGGGCC), two copies of the telo‐box (TBX: AAACCCT) and one starch box (SBX: AAGCCC) (Figure 5E) (Michael et al., 2008). It will thus be worthwhile in future to express *AtCAB2p::LUC+* in *K. laxiflora* and determine whether the phase shifts to match that demonstrated here for the native *KlCAB2p*.

The free‐running period of both *AtCAB2p* and *KlCAB2p* in Arabidopsis was similar in mature leaves to the established endogenous free‐running period of ~24‐h. Whether periods for *KlCAB2p* increased with leaf age in Arabidopsis, as observed for *KlCAB2p* in *K. laxiflora*, was less clear as fewer LUC+ datasets from individual leaves were found to be rhythmic. The data for *AtCAB2p* displayed a trend of period lengthening as new leaves appeared. These results were consistent with those presented by Kim et al. (2016) using Arabidopsis. Importantly, these results raise key questions about the differences between the two species, and the evolution of their circadian biology across the respective leaf developmental profiles.

Overall, this study demonstrated that the firefly LUC reporter gene system can be used as a robust, high‐throughput and reproducible experimental tool for understanding the molecular‐genetic basis for the circadian rhythms of CAM‐associated genes in the model species *K. laxiflora*. Crucially, by comparing promoter activity in CAM leaves of *K. laxiflora* to the activity of the same *p::LUC+* construct in C_3_ Arabidopsis, differences were discovered between clock control and timing of *KlCAB2p* relative to the light/dark cycle. Future studies should dissect the mechanistic basis for the interaction between the core‐clock of Arabidopsis and promoter regions from *K. laxiflora*. Such studies will shed light on the evolution of clock‐control of CAM, perhaps revealing at the level of individual promoter motifs the adoption of stress responsive elements from C_3_ species. Despite the progress presented in this study, there remains a pressing need for a much more detailed exploration of the individual clock‐controlled promoter motifs within CAM gene promoters, and the cognate clock‐controlled TFs that bind to and regulate CAM gene promoters. Understanding such interactions and signalling networks better would ultimately allow for temporal control to be optimised when considering engineering a drought‐inducible CAM system into C_3_ crops.

## METHODS

### Plant materials

The *K. laxiflora* Baker plants used for the generation of transgenic lines were originally obtained from the University of Oxford Botanic Gardens (OBG), UK. Ploidy measurements using flow cytometry determined this accession is diploid with an estimated genome size of 274 Mb, and self‐pollination trials established the ability of this line to set viable seed. The seed used here for stable transformation was the progeny of three rounds of self‐pollination and single seed descent.

For seedling growth and regeneration of transgenic plants via tissue culture, seeds were surface sterilised with ethanol and germinated on ½ × Murashige and Skoog (MS) medium with Gamborg's B‐5 vitamins (Duchefa, Haarlem, The Netherlands) and 3% (w/v) sucrose (Sigma, Welwyn Garden City, UK) containing 0.8% (w/v) Phytoagar (Duchefa). Plates of seedlings were cultured in walk‐in growth chambers set to provide 16‐h‐light/8‐h‐dark at a constant temperature of 22°C. Mature transgenic lines and wild type *K. laxiflora* were grown in compost mix 1:1:1:3 M2 (Multi‐Purpose Compost, Levington M2): John Innes (Sinclair's John Innes No. 2 Compost): Sinclair compost (Sinclair Potting compost): Perlite (Sinclair Perlite Standard) with additional nutrients from Osmocote beads (ICL Osmocote Pro Slow‐Release Fertiliser 5‐6), added according to manufacturer's instructions. Mature plants were grown in glasshouses with a minimum temperature of 20°C and supplementary lighting from sodium metal halide lamps ensuring a year‐round minimum daylength of 16‐h‐light/8‐h‐dark. Plants were entrained for circadian free‐running experiments using Snijders Microclima MC‐1000 plant growth cabinets set to 12‐h‐light (450 μmol photons m^−2^ sec^−1^), 25°C, 60% humidity/12‐h‐dark, 15°C, 70% humidity.

Arabidopsis plants of ecotype Col‐0 were grown at constant 22°C with 16‐h‐light/8‐h‐dark in glasshouses and plant growth rooms. For selection of transformants, Arabidopsis seeds were surfaced sterilised using 70% ethanol and plated to germinate on ½ × MS medium with Gamborg's B‐5 vitamins, 0.8% (w/v) Phytoagar and 15 μg ml^−1^ hygromycin B (Duchefa).

### Cloning of *KlGPT2p* , *KlCAB2p* and *KlPPCK1p* regions and construction of *p::LUC+* binary vectors

Putative promoter regions of ~3000 bp were selected upstream of the candidate 5′ transcription start site (TSS) of the target gene and included the 5′UTR up to the predicted ATG start codon. For *KlGPT2p* and *KlPPCK1p*, cloning primers were designed against the tetraploid ‘*Kalanchoë laxiflora* v1.1’ genome available on the JGI Phytozome database, with the accession numbers Kalax.0007s0180.1 (*KlGPT2*) and Kalax.0021s0061.1 (*KlPPCK1*). *KlCAB2p* was amplified and cloned from the diploid *K. laxiflora* OBG accession that was used to generate all the transgenic lines.

Each ~3000 bp promoter fragment was amplified from wild type *K. laxiflora* genomic DNA using gene‐specific primers with Gateway™ ready attB recombination sites added as 5′ extensions. These primer extensions allowed the resulting PCR products that were amplified with KOD Hot‐Start DNA polymerase to be recombined into the pDONR201 Gateway™ entry vector using a BP Clonase recombination reaction according to the manufacturer's instructions (Thermo Fisher Scientific, Loughborough, UK). Each *Kalanchoë* promoter insert in a pDONR201 entry clone was sequenced in full using Sanger sequencing (Eurofins, Wolverhampton, UK) to confirm the correct sequence and orientation. Standard Gateway^®^ cloning was carried out to recombine promoter regions into the *pPCV_LUC+_GW* binary vector (GenBank Accession number: PQ770919) (Katzen, 2007). Plasmids were transformed into competent cells of *A. tumefaciens* strain GV3101 pMP90RK using electroporation, and selected on LB‐agar plates containing rifampicin 50 μg ml^−1^, gentamycin 25 μg ml^−1^, kanamycin 50 μg ml^−1^ and carbenicillin 100 μg ml^−1^.

### Stable transformation of *K. laxiflora* diploid accession OBG using *A. tumefaciens* and tissue culture

*Kalanchoë laxiflora* tissue from 4‐ to 6‐week‐old seedlings germinated and grown in sterile conditions was used for generation of all stable transgenic lines. Roots were removed from plantlets and leaves were cut into small explants approximately 5 mm wide using a sterile scalpel in a laminar flow bench. Explants were submerged in the appropriate strain of *A. tumefaciens* GV3101 pMP90RK harbouring the desired *pPCV_LUC+_GW* binary vector for 1 h and blotted on sterile filter paper before being transferred to co‐cultivation plates. The regeneration of callus and plantlets was carried out as described by Dever et al. (2015), with the exception of the antibiotics used. Hygromycin selection at working concentration 5 μg ml^−1^ was used to select for successful transformation of plant material through the presence of *pPCV_LUC+_GW p::LUC+* binary vector, and Timentin at 300 μg ml^−1^ was used to prevent over‐growth of *Agrobacterium* (Wang et al., 2019).

Once plantlets with roots were regenerated, they were transferred into soil. They were grown first in plugs ~5 cm wide until they measured ~10 cm tall and had established a substantial root network. Plants were then transferred to 12 × 12 cm pots and grown to a height of ~30 cm prior to their entrainment to 12‐h‐light/12‐h‐dark conditions in preparation for imaging experiments.

### Floral dip transformation of Arabidopsis

Flowering plants of *Arabidopsis thaliana* ecotype Col‐0 that were aged 4–6‐weeks were dipped once in the appropriate liquid culture suspension of *A. tumefaciens* transformed with the desired *pPCV_LUC+_GW_p::LUC+* construct. The *A. tumefaciens* cells were grown overnight in 250 ml LB medium. They were then mixed directly with 250 ml 2× concentration infiltration media to give a final concentration of 10% (w/v) sucrose, 0.025% (v/v) Silwett L‐77. Post‐dip, plants were watered until the first siliques began to dehisce and then allowed to dry for ~3 weeks, at which point the seed was harvested and cleaned (Clough & Bent, 1998).

T0 seeds were selected on 15 μg ml^−1^ hygromycin according to protocol by Harrison et al. (2006). Resulting T1 plants were genotyped using diagnostic PCR amplification of a ~400‐bp fragment that spanned the junction region between the 3′ end of each *K. laxiflora* promoter and the 5′ end of the open reading frame of the *LUC+* gene. T2 seeds were collected from positive clones and used in imaging experiments at 7‐days after vernalisation in the cold for at least 48‐h.

### LUC imaging of transgenic lines and image analysis

Plants were entrained in 12‐h‐light/12‐h‐dark for 7 days prior to imaging, with growth chamber conditions as described above. To set up the imaging plates for *K. laxiflora*, LP1–LP6 were excised from each plant at their petiole using a scalpel 18–24 h before the start of each experiment, and half the leaf and the petiole was then removed, retaining the mid‐rib. Side‐by‐side imaging experiments using intact detached leaves and the half leaves retaining their mid‐rib demonstrated that cutting each leaf in half did not change the properties of the circadian rhythms of LUC+ activity that were recorded (Figure S5). Each leaf explant was then placed on plates containing 0.4% agar dissolved in distilled water (pH 5.8). They were sprayed evenly with a solution containing filter‐sterilised 5 mM luciferin in 0.01% (v/v) Triton X‐100 in distilled water. Plates with leaves were then returned to the growth cabinet under 12‐h‐light/12‐h‐dark entrainment conditions for the following ~18 h, until the start of the next light period.

For each time course imaging of LUC bioluminescence, plates of pre‐entrained, excised leaves were transferred to the imaging cabinet set to a constant light intensity of ~30 μmoles photons m^−2^ sec^−1^ and a constant temperature of 15°C. Illumination was provided by a mix of red and blue LEDs as described previously (Boxall et al., 2017, 2020; Gould et al., 2009). The software ‘micromanager’ connected to a programmable Arduino computer was used to control a Retiga LUMO™ CCD camera. Imaging commenced at the time that would have been the start of the 12‐h‐light period during pre‐entrainment. Lights were switched off before each exposure of the camera, which lasted 20 min. Images were captured every 2‐h spanning a period of ~7 days under constant light and temperature (LL), circadian free‐running conditions.

### Data analysis of LUC+ imaging measurements and statistical analysis of circadian rhythmicity and period length

Image stacks were analysed in Fiji (Schindelin et al., 2012), selecting a small consistent region of the leaf towards the apex to the side of the midvein, and measuring the ‘mean grey value’ of pixels as a quantitative measure of bioluminescence. Background light levels, measured away from the leaves, were quantified in the same way and subtracted to normalise data across each time course of images.

For circadian analysis and statistics, including rhythmicity and period estimates, Biodare2 (www.biodare2.ed.ac.uk) was used (Moore et al., 2014; Zielinski et al., 2014). Period was calculated using linear detrending and fast Fourier transform‐non‐linear least squares and then compared to spectral resampling (SR) to ensure validity of results. Rhythmicity was also determined using Biodare2. Data were filtered for non‐rhythmic replicates prior to period calculations (Tables S1–S4).

## CONFLICT OF INTEREST

The authors declare that they have no conflicts of interest.

## Supporting information


**Figure S1.** The *KlPPCKp::LUC+* construct did not drive rhythms of LUC+ activity in four independent transgenic lines of *K. laxiflora*.

**Figure S2.** *Kalanchoë laxiflora* accession OBG diploid displayed a robust and persistent circadian rhythm of CAM‐associated CO_2_ exchange under constant light and temperature (LL) free‐running conditions.

**Figure S3.** Time lapse movies showing LUC+ activity over ~1 week in multiple replications of LP1–LP6 of *K. laxiflora* for the genotypes *KlCAB2p::LUC+* (left) and *KlGPT2p::LUC+* (right). Images were captured every 2‐h for ~7‐day under LL at 15°C. Video can also be accessed at https://youtu.be/HdpArmDxA6M?si=z_H5GIqJ7U2C1Sjr.

**Figure S4.** *KlCAB2p* drives low amplitude expression of consistent phasing in Arabidopsis, independently of *AtCAB2::LUC+*.

**Figure S5.** Rhythms of LUC+ bioluminescence generated by half leaf explants can recapitulate those of intact detached leaves of *K. laxiflora*.

**Figure S6.** Phasing of *KlCAB2p::LUC+* and *KlGPT2p::LUC+* changes throughout *K. laxiflora* leaf development.

**Table S1.** Statistics for Benjamin–Hochberg tests on LL free‐running rhythms for *KlCAB2p::LUC+* expressed in *K. laxiflora* line 19.
**Table S2.** Statistics for Benjamin–Hochberg tests on LL free‐running rhythms for *KlGPT2p::LUC+* expressed in *K. laxiflora* line N/2.
**Table S3.** Statistics for Benjamin–Hochberg tests on LL free‐running rhythms for *AtCAB2p::LUC+* (Label column AtCAB1.1, AtCAB1.2, etc. denote the biological replicates) and *KlCAB2p::LUC+* expressed in *A. thaliana* lines 1, 4, 7, 8 and 14 assayed using 7‐day‐old seedlings.
**Table S4.** Statistics for Benjamin–Hochberg tests on LL free‐running rhythms for *AtCAB2p::LUC+* and *KlCAB2p::LUC+* expressed in *A. thaliana* assayed using single detached leaves of different developmental ages.

## Data Availability

All relevant data can be found within the article and its Supporting Information, or can be requested from the corresponding authors.
