# Supplementary material for: A PROMOTER::LUCIFERASE reporter system reveals key elements of the circadian regulation of Crassulacean acid metabolism (CAM) in Kalanchoë laxiflora Baker
Source: Plant J. 2026 Jun 4;126(5):e70937. doi: 10.1111/tpj.70937 (PMC13238310; doi:10.1111/tpj.70937)
Supplement: Supplementary file 4 — Figure S4. KlCAB2p drives low amplitude expression of consistent phasing in Arabidopsis, independently of AtCAB2::LUC+. [file TPJ-126-0-s004.pptx]

## Slide 1
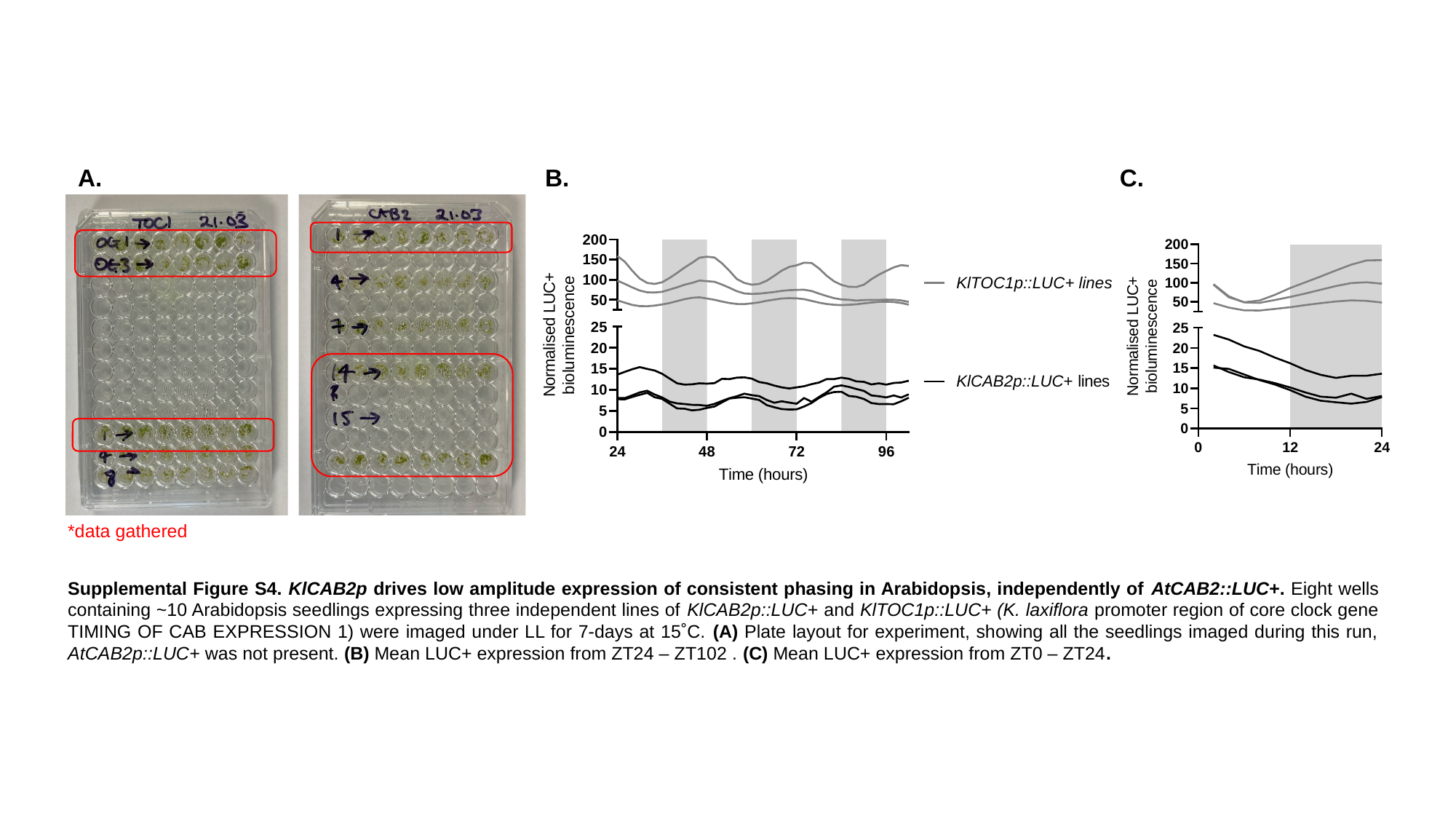

A. B. C.
*data gathered
Supplemental Figure S4. KlCAB2p drives low amplitude expression of consistent phasing in Arabidopsis, independently of AtCAB2::LUC+. Eight wells containing ~10 Arabidopsis seedlings expressing three independent lines of KlCAB2p::LUC+ and KlTOC1p::LUC+ (K. laxiflora promoter region of core clock gene TIMING OF CAB EXPRESSION 1) were imaged under LL for 7-days at 15˚C. (A) Plate layout for experiment, showing all the seedlings imaged during this run, AtCAB2p::LUC+ was not present. (B) Mean LUC+ expression from ZT24 – ZT102 . (C) Mean LUC+ expression from ZT0 – ZT24.
