# Supplementary material for: A PROMOTER::LUCIFERASE reporter system reveals key elements of the circadian regulation of Crassulacean acid metabolism (CAM) in Kalanchoë laxiflora Baker
Source: Plant J. 2026 Jun 4;126(5):e70937. doi: 10.1111/tpj.70937 (PMC13238310; doi:10.1111/tpj.70937)
Supplement: Supplementary file 5 — Figure S5. Rhythms of LUC+ bioluminescence generated by half leaf explants can recapitulate those of intact detached leaves of K. laxiflora. [file TPJ-126-0-s005.pptx]

## Slide 1
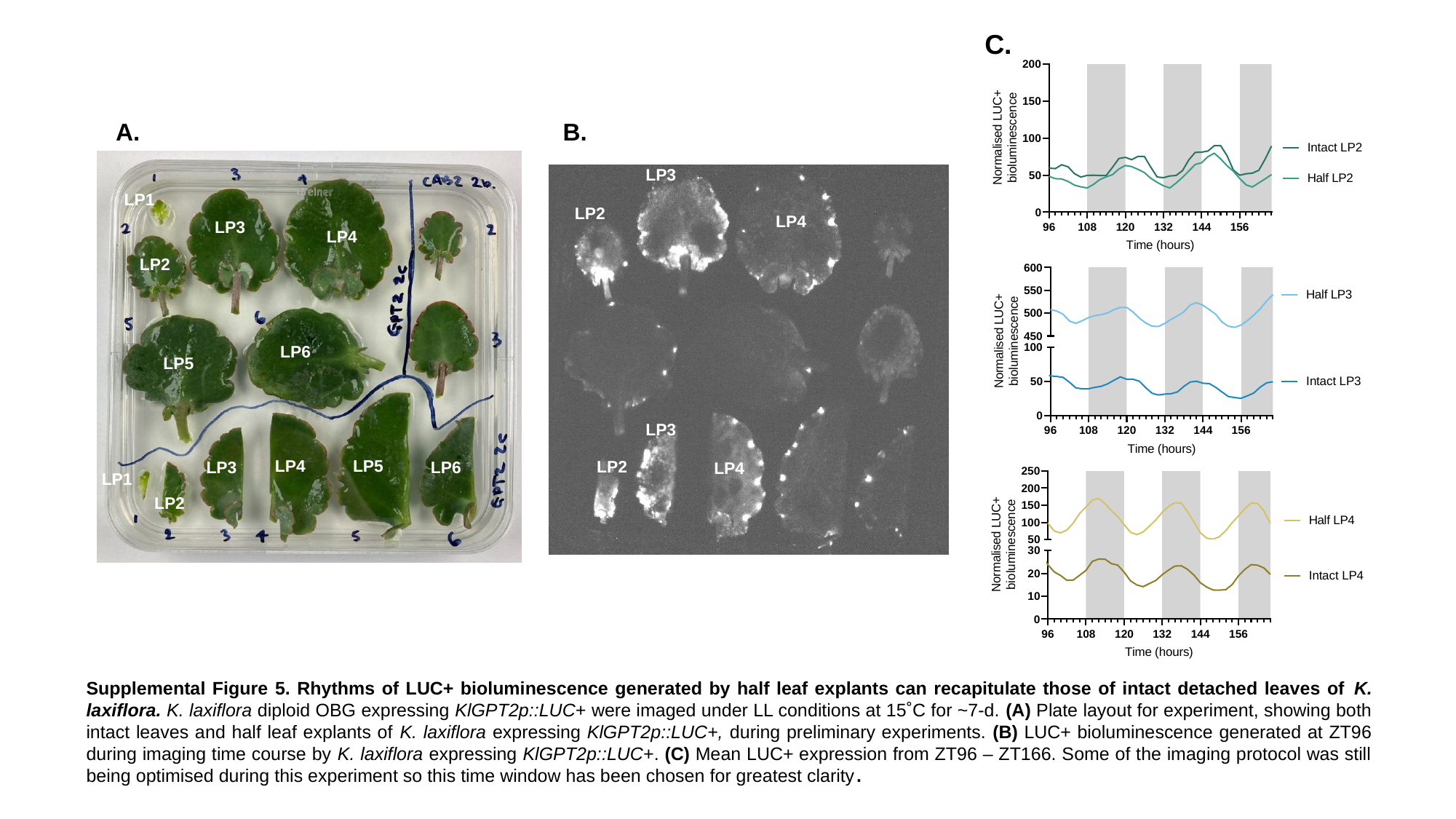

C.
LP1
LP3
LP4
LP2
LP6
LP5
LP4
LP5
LP3
LP6
LP1
LP2
LP3
LP2
LP4
LP3
LP2
LP4
A. B.
Supplemental Figure 5. Rhythms of LUC+ bioluminescence generated by half leaf explants can recapitulate those of intact detached leaves of K. laxiflora. K. laxiflora diploid OBG expressing KlGPT2p::LUC+ were imaged under LL conditions at 15˚C for ~7-d. (A) Plate layout for experiment, showing both intact leaves and half leaf explants of K. laxiflora expressing KlGPT2p::LUC+, during preliminary experiments. (B) LUC+ bioluminescence generated at ZT96 during imaging time course by K. laxiflora expressing KlGPT2p::LUC+. (C) Mean LUC+ expression from ZT96 – ZT166. Some of the imaging protocol was still being optimised during this experiment so this time window has been chosen for greatest clarity.
