# Supplementary material for: A PROMOTER::LUCIFERASE reporter system reveals key elements of the circadian regulation of Crassulacean acid metabolism (CAM) in Kalanchoë laxiflora Baker
Source: Plant J. 2026 Jun 4;126(5):e70937. doi: 10.1111/tpj.70937 (PMC13238310; doi:10.1111/tpj.70937)
Supplement: Supplementary file 6 — Figure S6. Phasing of KlCAB2p::LUC+ and KlGPT2p::LUC+ changes throughout K. laxiflora leaf development. [file TPJ-126-0-s007.pptx]

## Slide 1
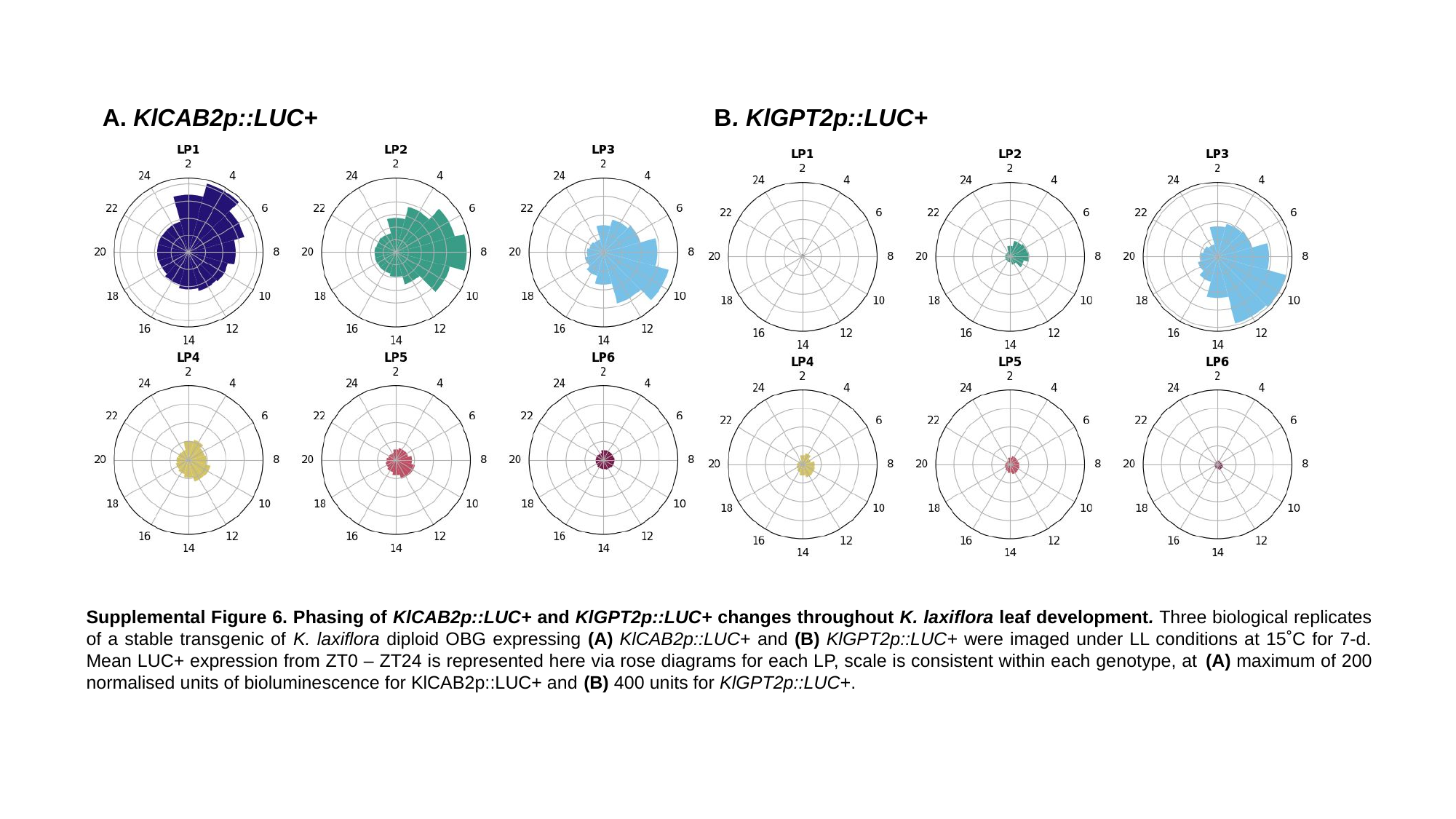

A. KlCAB2p::LUC+ B. KlGPT2p::LUC+
Supplemental Figure 6. Phasing of KlCAB2p::LUC+ and KlGPT2p::LUC+ changes throughout K. laxiflora leaf development. Three biological replicates of a stable transgenic of K. laxiflora diploid OBG expressing (A) KlCAB2p::LUC+ and (B) KlGPT2p::LUC+ were imaged under LL conditions at 15˚C for 7-d. Mean LUC+ expression from ZT0 – ZT24 is represented here via rose diagrams for each LP, scale is consistent within each genotype, at (A) maximum of 200 normalised units of bioluminescence for KlCAB2p::LUC+ and (B) 400 units for KlGPT2p::LUC+.
