# Supplementary material for: A PROMOTER::LUCIFERASE reporter system reveals key elements of the circadian regulation of Crassulacean acid metabolism (CAM) in Kalanchoë laxiflora Baker
Source: Plant J. 2026 Jun 4;126(5):e70937. doi: 10.1111/tpj.70937 (PMC13238310; doi:10.1111/tpj.70937)
Supplement: Supplementary file 7 — Table S1. Statistics for Benjamin–Hochberg tests on LL free‐running rhythms for KlCAB2p::LUC+ expressed in K. laxiflora line 19. Table S2. Statistics for Benjamin–Hochberg tests on LL free‐running rhythms for KlGPT2p::LUC+ expressed in K. laxiflora line N/2. Table S3. Statistics for Benjamin–Hochberg tests on LL free‐running rhythms for AtCAB2p::LUC+ (Label column AtCAB1.1, AtCAB1.2, etc. denote the biological replicates) and KlCAB2p::LUC+ expressed in A. thaliana lines 1, 4, 7, 8 and 14 assayed using 7‐day‐old seedlings. Table S4. Statistics for Benjamin–Hochberg tests on LL free‐running rhythms for AtCAB2p::LUC+ and KlCAB2p::LUC+ expressed in A. thaliana assayed using single detached leaves of different developmental ages. [file TPJ-126-0-s002.zip › tpj70937-sup-0008-Table_S1-S4_captions.docx]

Supplemental Table S1: Statistics for Benjamin-Hochberg tests on LL free-running rhythms for *KlCAB2p::LUC+* expressed in *K. laxiflora* line 19. Tests were for three biological replicates of leaf pair 1 (LP1), LP2, LP3, LP4, LP5 and LP6 (e.g. Labels 1.1, 1.2 and 1.3 denote the data for the three biological replicates for LP1).

Supplemental Table S2: Statistics for Benjamin-Hochberg tests on LL free-running rhythms for *KlGPT2p::LUC+* expressed in *K. laxiflora* line N/2. Tests were for three biological replicates of leaf pair 1 (LP1), LP2, LP3, LP4, LP5 and LP6 (e.g. Labels 1.1, 1.2 and 1.3 denote the data for the three biological replicates for LP1).

Supplemental Table S3: Statistics for Benjamin-Hochberg tests on LL free-running rhythms for *AtCAB2p::LUC+* (Label column AtCAB1.1, AtCAB1.2, etc denote the biological replicates) and *KlCAB2p::LUC+* expressed in *A.thaliana* lines 1, 4, 7, 8 and 14 assayed using 7-day-old seedlings. For each independent *KlCAB2p::LUC+* line (1, 4, 7, 8 or 14) the biological replicates are denoted 1.1, 1.2, 1.3 …..1.8_.

Supplemental Table S4: Statistics for Benjamin-Hochberg tests on LL free-running rhythms for *AtCAB2p::LUC+* and *KlCAB2p::LUC+* expressed in *A.thaliana* assayed using single detached leaves of different developmental ages. Note that leaves denoted “1.1” were the oldest leaves and “4.1” the youngest. Each leaf age was assayed across 9 biological replicates, denoted 1.1_1, 1.1_2, 1.1_3 etc in the Label column. For *KlCAB2p::LUC+* expressed in *A.thaliana,* independent line 4 was used; hence the Labels have the syntax KlCAB2_4_1.1_1, KlCAB2_4_1.1_2, KlCAB2_4_1.1_3 etc.
